# Coercive Public Health Policies Need Context-Specific Ethical Justifications

**DOI:** 10.1007/s40592-024-00218-x

**Published:** 2024-10-15

**Authors:** Tess Johnson, Lerato Ndlovu, Omolara Baiyegunhi, Wezzie Lora, Nicola Desmond

**Affiliations:** 1 Ethox Centre, Nuffield Department of Population Health, University of Oxford, Oxford, UK; 2 Pandemic Sciences Institute, Nuffield Department of Medicine, University of Oxford, Oxford, UK; 3 Africa Health Research Institute, Durban, South Africa; 4 University of KwaZulu-Natal. Durban, South Africa; 5 University Alabama Birmingham, Birmingham, USA; 6 Department of Public Health, Malawi Liverpool Wellcome Programme, Blantyre, Malawi; 7 Liverpool School of Tropical Medicine, Liverpool, UK

## Abstract

Public health policies designed to improve individual and population health may involve coercion. These coercive policies require ethical justification, and yet it is unclear in the public health ethics literature which ethical concepts might justify coercion, and what their limitations are in applying across contexts. In this paper, we analyse a number of concepts from Western bioethics, including the harm principle, paternalism, the public interest, and a duty of easy rescue. We find them plausible justifications for coercion in theory, but when applied to case studies, including HIV testing in Malawi, vaccine mandates in South Africa, and prohibitions of antibiotic use in livestock in the EU, their limitations become clear. We argue that the context-specificity of ethical justifications for coercion has been overlooked, and there is more work needed to identify context-relevant ethical justifications for coercive policies in various settings and for various populations, rather than relying on universalising Western bioethical justifications across all contexts.

## Introduction

Health is often viewed as part of the private sphere—my health is my business, and your health is your business; we make individual choices concerning whether to protect and promote our own health or not. However, in reality, health is highly interdependent between individuals, at the community level, and across populations—my health, it seems, is not only my own business. Population-wide health is influenced by a combination of environmental, structural and biological factors. Additionally, an individual’s healthcare choices can impact others, particularly if they come into contact or if they influence each other’s behaviour. Actions for health protection and promotion are key among the responsibilities of national governments (Frieden 2013; Resnik 2007). Public health policies are designed to improve individual and population health through a range of interventions that may involve education, incentivisation, compensation, disincentivisation, or mandatory/compulsory health actions, among others (Nuffield Council on Bioethics 2007).

Thus, certain policies may result in or involve, either directly or indirectly, elements of coercion. For instance, mandatory or compulsory preventive measures imposed by states are clearly coercive in a direct way. Public health policies, however, may also result in coercion indirectly where they influence behaviours and interactions such that people or groups coerce each other, without direct state intervention. An educational policy, for example, may raise awareness of the need for treatment for a certain disease; a church policy may enforce ‘voluntary’ couples HIV testing as a prerequisite for conducting a marriage. These may then result in individuals coercing each other into getting treated or tested.

Coercive policies present a potential problem for policymakers, at least as framed in Western bioethics traditions which often hold a presumption in favour of liberty (Feinberg 1984).^2^ According to this presumption, it is better to not infringe on people’s freedoms, all else equal. In order to justify the infringements of liberty that may be involved in coercing people via a particular policy, there must be moral reasons in favour of coercion that outweigh the moral significance of liberty. In the public health sphere, there may be existing coercive policies that are inadequately justified—either because the ethical concepts grounding coercion in that context are not clear, or because they are not weighty enough to justify coercion. Outside of Western bioethics traditions, examining the ethical justifications behind coercive policies is also important for ethically reflective policymaking, but ethical analysis may not be driven by the same Western presumption in favour of liberty. Instead, framings from other ethical traditions around the world may emphasise the need to reflect on coercive policies for other reasons. For instance, in contexts where Ubuntu ethics more appropriately guides policy, it may be necessary to reflect upon coercive policies through concepts of reciprocity, common good, and peaceful relations (Ujomudike 2016). Insofar as coercive public health policies may be less likely to promote peaceful relations or reciprocity, they may be particularly in need of ethical analysis.

It is outside the scope of this work to consider coercion from all ethical traditions. Rather, we highlight *why* it is important to do so, by critiquing applications of Western bioethical concepts in ethical analysis of coercive policies across global contexts. In doing so, we analyse both the limitations *within* Western bioethical concepts that might justify coercive public health policies, and, more briefly, the limitations regarding when the concepts should be considered to begin with. The broader purpose of this work is to explore instances where coercion is or is not justified in public health policy, and thus, when alternative policies should be considered. We first introduce the concept of coercion, then outline examples of public health policies that involve coercion. By using case studies of coercive policies implemented in various global contexts with different geographies, economies, health systems, social and cultural norms, moral frameworks, and political systems, including Malawi, South Africa, and the United Kingdom, we highlight how the strength and applicability of many of these justifications is context-specific.

## 1 Coercion in Public Health Policy

In the Western philosophical literature, a significant divide exists between ideas of coercion that are essentially moralised, and those that are neutral (Zimmerman 2002). Where coercion is moralised, it might be conceived as intrinsically and necessarily wrong in that it involves an agent, *P*, harming or otherwise wronging the target, *Q* (Anderson 2021). Here, we employ a definition that is mostly descriptive rather than moralised, but we acknowledge that there may be reasons to avoid coercive action. Descriptively, coercion occurs when a person *P* aims to keep another, *Q*, from performing a particular action by employing a credible threat of what *P* will do to *Q* if *Q* does the action. This is based on Robert Nozick’s work on coercion (1969).

Nozick’s definition contains some normativity, implied by the use of the loaded word ‘threat’. Additional normativity is added if we follow Joel Feinberg in supporting a presumption in favour of liberty (1984). The result is that the burden of proof for justifying coercion is on those who would have it enacted through public policy.

Public health is a unique area of public policy where coercion often involves state bodies compelling individuals or groups to adopt healthy behaviours that contribute to protecting crucial healthcare resources. Most often, the targets of coercion will be groups, as a core aim of public health is to improve outcomes at the population scale, and as it is impractical to make policy specific to individuals. So, there might be blanket cases where a whole population is mandated to get a certain vaccine (with some exclusions), or where all doctors are required avoid prescribing antibiotics for mild and self-limiting infections. These kinds of actions involve liberty restrictions. Parents in Australia, for example, are not allowed to reject vaccinations on behalf of their children. If they do so, any national welfare payments or subsidies for childcare costs that they receive are stopped (Attwell and Drislane 2022). A policy like this restricts parents’ freedom to deny medical treatment. The presumption in favour of liberty demands that coercion like this be justified in some way, to render the policy ethically acceptable. Justification for a coercive policy like this might refer to the prevention of harm. Unvaccinated children pose a harm to others, because they can be vectors for the spread of infectious diseases to others (or at least, others who cannot receive vaccinations due to allergies, medical vulnerabilities, etc.). To prevent some children harming others, the state may need to coerce them (or, technically, their parents, as those providing proxy consent for the vaccinations, and as those who suffer the consequences if the vaccination schedule is not complied with) via public health policy. This justification, however, only holds where there is a risk of harm to others from non-vaccination, and where the children being vaccinated are not seriously harmed. This example demonstrates the complexity behind seemingly straightforward and acceptable coercive public health policies, even where a Western bioethical concept is used as a justification in a Western policy context.

Having provided an initial idea of coercion and how it might feature in the sphere of public health, we now turn to examining the possible ethical concepts that might be used to justify coercion.

## 2 Ethical concepts that might be used to justify coercion

In this section, we outline ethical concepts in the Western tradition that might counterweigh the presumption in favour of liberty, thus justifying coercive public health policies in a manner appropriate to Western contexts. What we intend here is not a systematic review but rather a focus on concepts most commonly used to ethically justify coercion, and that have previously been (perhaps inappropriately) applied in other global contexts to justify policy decisions, as well. Many of these concepts are derived from Feinberg’s discussion of coercion-legitimising principles, including: public or private harm (or risk of harm); legal paternalism; legal moralism; the offence principle; moralistic paternalism; and the benefit-to-others principle. In particular, the harm principle, public interest, paternalism and the duty of easy rescue are common adaptations of some of these concepts as they are referred to within the public health ethics literature. We discuss each of these below.

We do not include other possible justifications that feature in the literature, such as reasons for coercion based on health justice (Reid 2020), duties based on moral responsibility (Johnson and Matlock 2022), application of the precautionary principle (Nijsingh et al. 2020), considerations of community-level reciprocity under the Ubuntu tradition (Ujomudike 2016), or others (Johnson 2024a). This is not because these concepts cannot provide a strong justification for coercive public health policy, but rather because we have chosen concepts that have been prominently discussed in Western bioethical tradition and applied across global contexts, to highlight the limitations of concepts that may have (had) the most effect on policymaking.

### The harm principle

In *On Liberty*, J.S. Mill introduces a harm principle that states that preventing harm to others is the only possible justification for coercion: “That principle is, that the sole end for which mankind are warranted, individually or collectively, in interfering with the liberty of action of any of their number, is self-protection.” (Mill 1859, 1.1). By ‘liberty of action’, Mill means basic liberties including freedom of speech, conscience, life plans, and association. By ‘harm’, he intends a setback to important interests, distinguishing this from ‘mere offense’ (Mill 1859, 1.4).

This short passage has received significant attention in discussions of coercion, and has sometimes, mistakenly, been used as a definitive, positive justification. However, Mill makes it clear that prevention of harm is a necessary, but potentially insufficient condition to justify restricting liberty. To build the harm principle into a necessary and sufficient condition for justified coercion, more is needed. Joel Feinberg uses a discussion of criminal law to evidence his positive claim, that “It is always a good reason in support of penal legislation that it would probably be effective in preventing (eliminating, reducing) harm to persons other than the actor (the one prohibited from acting) and there is probably no other means that is equally effective at no greater cost to other values.” (1984, 27) This provides a positive justification. He adds caveats to this, such that the harm in question must be substantial and avoidable. The substantiality requirement might be considered both in absolute terms, akin to Mill’s distinction between harm and mere offence, and in comparative terms, with Feinberg’s stipulation that “only the prevention of still more serious harms to others could justify its infliction [through legal coercion].” (1984, 12)

In bioethics, the harm principle or more broadly a ‘Millian paradigm’ centring on the principle has been thought to underpin much public health policymaking in a number of countries worldwide (Coggon 2008). This justification has been referred to in relation to public health interventions including social distancing during the COVID-19 pandemic. The argument is that mandatory social distancing is a justified deprivation of liberty on the basis that those who fail to stay away from others during a pandemic risk transmitting disease and causing serious illness in others, which the state is ethically justified in preventing (Matose and Lanphier 2020). However, in reality, the strength of this justification and competing reasons against social distancing may differ according to context-specific factors, as we will explore in our final discussion.

In many cases of coercive public health policies, it can be difficult to separate out a policy that aims to prevent harm to others from one that aims to prevent harm to the actor, and thus to separate out justifications from the harm principle *vs* from paternalism. Another clear-cut case of the harm principle as justifying coercive public health policy considers surveillance of healthy people who are carriers of drug-resistant pathogens that might pose risk to others (Jamrozik and Selgelid 2020). The claim is that if resistant strains are identified that the healthy carriers might transmit to others, harming those others, then there seems to be an initial justification for surveillance and subsequent coercive measures, such as “reporting the diagnosis of asymptomatic carriage to authorities, notification of third parties, monitoring of carriers, restrictions on freedom of movement (e.g., quarantine, isolation, travel bans), exclusion of carriers from working in certain occupations, and/or possibly even requirements for treatment of carriers in certain circumstances” (2020, 185). These must all be justified by the harm principle, and not paternalism, as the health of the asymptomatic carriers is not at stake. This is commonly the case, as most resistant pathogens are hosted by asymptomatic carriers, as the authors discuss. It is only when potential harms to others are considered that we find a possible justification for coercion in this context.

### Paternalism

Mill’s stipulation of the harm principle excludes restrictions upon liberty as justified by protecting the individual from harming themselves. Concerning “the way of compulsion and control, whether the means used be physical force in the form of legal penalties, or the moral coercion of public opinion”, Mill holds that “[An individual] cannot rightfully be compelled to do or forbear because it will be better for him to do so, because it will make him happier, because, in the opinions of others, to do so would be wise, or even right.” (Mill 1859, 1.1). That is, prevention of harm to others may justify intervention, but prevention of harm to self cannot (Conly 2014). Mill’s reasons for rejecting paternalistic justifications for third-party intervention come down to the potential for states to abuse their power in the name of protecting their citizens, and the mis-identification of the good for others.

Feinberg, by contrast, allows paternalistic justifications for coercion, separating out the typical conception of paternalism (which he labels ‘legal paternalism’) from the prevention of *moral* harm to the person, which he terms ‘moralistic paternalism’ (1984, 13). Whilst the prevention of *moral* harm to self or others rarely features in policy discussions today as a principle limiting the law (Harcourt 1999), perhaps due to having been used in state abuses of power, debates about whether ‘legal’ paternalism can justify coercive policy continue. Ronald Dworkin holds that paternalism is justified under two conditions. First, it must protect against someone’s irrational tendencies, and second, it must only concern actions that are “far-reaching, potentially dangerous, and irreversible” (Dworkin 1971). This provides only a highly limited scope for paternalistic action, in contrast with the view that paternalism is justified in a broader range of cases, especially when pertaining to preventing groups from harming themselves (Beauchamp 1985).

In the public health ethics literature, key policy interventions that have been discussed include seatbelt mandates (Conly 2014), making sugar-sweetened beverages less accessible (Barnhill 2019), and criminalisation of suicide and/or euthanasia (Hands 2009). As James Wilson argues (2011), paternalistic justifications may be particularly forceful in public health. One of the main objections to paternalism is that the individual is the best judge of their own good. However, health is an uncontroversial good, so it seems that protecting and promoting it will, in the vast majority of cases, be good for the individual. Protecting an individual from severe injury in a car accident by mandating seatbelts or reducing their likelihood of suffering from diabetes later in life by taxing sugar-sweetened beverages are highly likely to benefit the individual, overall. What’s more, they remove choices that are, on the whole, unlikely to be important to the individual’s leading a good life according to most theories of wellbeing, such as the choice not to wear a seatbelt, or the choice to drink a sugary beverage. That said, the validity of paternalism may vary significantly according to the moral framework of a population, the legitimacy of a paternalistic state, and other context-specific factors, as we explore later.

### Public interest

Returning to Feinberg, he relies in part on a ‘benefit-to-others principle’ to justify infringements of liberty. He claims, “It is always a morally relevant reason in support of a proposed prohibition that it is probably necessary for the production of some benefit for persons other than the person who is prohibited.” (1984, 27) Feinberg does not provide much further discussion of this concept, but we see discussions of the public interest, and how this may justify coercive measures.

Before exploring these cases, it is important to note the difference between the harm principle and the public interest. Whilst the harm principle provides a strong justification based on avoiding making others worse off, the public interest provides a slightly weaker justification based on aiming to make others better off. It is commonly held in the literature that there is an asymmetry between harm and benefit, such that it is more important to avoid harm than to pursue benefit (Francis 2016). However, there may still be some extra force added to an argument for a coercive policy by reference to the benefits it produces for others.

Where Western ethics is often concerned with interferences with liberty, Western economics is sometimes concerned in parallel with interferences with the market. One primary justification for state interference is market failure—roughly, where the market fails to allocate goods to people in an optimal way. This commonly occurs in the production of public goods. Public goods are, by nature, non-excludable and non-rival, meaning that anyone can access them and no one’s benefiting from them prevents another’s benefit (Samuelson 1954). Because there is thus no incentive for the private provision of such goods, governments step in to provide and maintain them. Many public goods occur in the health sphere. A public healthcare system might be considered (in a limited way) a public good, as might free health education, screening and other interventions (Anomaly 2023). Some public health policies may also produce public goods – for instance, population vaccination campaigns might produce the public good of herd immunity, which boosts the population’s protection against an infectious disease (Giubilini 2019a). In cases like this, it seems that there is both an economic and an ethical reason based on the public interest to ensure such goods are secured – including via coercive policy. This has been argued in cases like vaccine mandates for particular populations in public health emergencies (Schaefer et al. 2022). However, it has also been argued against as insufficiently strong to justify coercion in cases such as taxing antibiotics in healthcare settings (Giubilini and Savulescu 2020). This argument may rely on the asymmetry between benefiting and harming, and may also rely on a comparison between the magnitude of benefit produced by a coercive public health policy in comparison to the harms it inflicts, the values of which harms and benefits may be judged differently by different populations facing different circumstances and threats around the globe.

### Duty of easy rescue

The final concept that has gained more attention recently as a justification for coercive public health measures in the bioethics literature is the duty of easy rescue. This concept does not have an analogue in the criminal law setting, at least as far as Feinberg is concerned. However, this concept, too, has more philosophical roots in Peter Singer’s duty of beneficence. Singer’s *Famine, Affluence and Morality* famously uses the thought experiment of a child drowning in a pond (1971). In this case, there is a clear duty to help the drowning child, if helping will come at little cost to oneself. This is equally the case, Singer holds, for those who are suffering far away, but who we can effectively help, at little cost to ourselves, via donations or other charitable help. The result is a duty of general beneficence, roughly that whenever we can assist someone at insignificant or reasonable personal expense, we should. However, the duty becomes less straightforward, as the individual cost increases. We might also argue that the duty weakens when the benefit to another is only small, or when the benefit is large in absolute terms, but small in comparison to a large cost imposed on the helping individual. Thus, there are limits to what constitutes a reasonable cost.

Julian Savulescu (2016), Alberto Giubilini (2019b), and others (Giubilini et al. 2018) have extended this duty to the collective context, particularly when considering our responsibility to benefit a group of people. They apply the argument to several public health policies. For instance, enforcing quarantine during a pandemic through coercive state action may be deemed justifiable. The justification arises from the underlying moral duty that individuals ought to take on this small cost to themselves in order to prevent serious harm (through the spread of a virulent infectious disease) to the population (Giubilini et al. 2018).

When it comes to addressing the issue of antimicrobial resistance, taxing antibiotics used by individuals to prevent or reduce the effects of mild and self-limiting infections is justified, even though the policy is coercive, according to the duty of easy rescue (Giubilini 2019b). The obligation in some cases holds because of the potential to benefit and/or reduce harm to others by protecting the antimicrobial commons and not consuming antibiotics. The moral reasoning holds true when the personal cost to the individual, which involves enduring a mild and self-limiting infection, is relatively low. However, it is important to recognise that in other settings, the ethical evaluation of public health policies may vary depending on the values and circumstances of the people compelled to comply. This may mean that the costs to individuals of complying with a certain policy may be considerably higher in different contexts. Therefore, the ethical justification of actions like antimicrobial stewardship or quarantine may be justified by the duty of collective easy rescue in some contexts, but not others.

Having explored several prominent ethical concepts that could serve as justifications for coercive policies in the realm of public health, we now shift our focus to how these concepts apply to three distinct public health policies: HIV testing in Malawi, vaccine mandates in South Africa, and antimicrobial stewardship in the European Union and UK. Each of these policies addresses significant health threats that are experienced worldwide, and yet they also present interesting contextual differences that highlight the limitations of the Western bioethics concepts introduced as potential justifications for coercion above in terms of the strength and applicability of those concepts. The implications of the context-specificity we highlight though these cases likely holds for other public health policies and other global contexts, and opens the floor for further discussion of how ethical justification for policies should be specific to the public health policy and the context where it is considered for implementation.

We turn now to the three case studies that we use to highlight ethical issues arising with coercive public health policies. These case studies have been chosen both for personal and illustrative reasons. Regarding the first reason, each of us has experience conducting research in at least one of these contexts, and our research on public health has led us to distinct questions regarding whether coercion is happening in that context, and whether it is justified. This theme of difficult questions surrounding the coercive nature of the policies brings the case studies together. Second, each of these three case studies illustrates a different structural context. In Malawi, the specifics of that policy jurisdiction, including mechanisms for HIV testing and treatment funding, the epidemiological context, social norms pertaining to pregnancy and motherhood, etc., render it different from any other context around the globe. The question of how coercion can be ethically justified in that context is, then, specific to the HIV testing in Malawi setting alone. Simply citing the harm principle or the duty of easy rescue is no guarantee that coercion is appropriate here (although it may be that some of these or other concepts do indeed apply well or may converge on the same judgement of a coercive public health policy). In South Africa, although the case of corporate vaccination requirements may mirror policies imposed in other settings like the United States of America, there are particularities concerning, for instance, South Africa’s history of mandatory vaccination against other diseases which affect the structures that inform whether a particular ethical justification applies, and which ethical justifications ought to be referred to, to begin with. In the United Kingdom, there are, again, features of this context that have allowed for particular actions concerning uses of antimicrobials in farming. The structures that may render a policy appropriate in the British context do not necessarily hold for others (even as nearby as France or Germany). Every policy jurisdiction has its own structural differences that mean that a specific set of ethical concepts ought to be considered in the justification of coercive action, and that some of the more commonly universalised Western bioethical concepts may not apply in that context.

## 3 Coercive policy and ethics in HIV testing

WL and ND conduct HIV research in Malawi and have played a significant role in introducing HIV self-testing (HIVST) in Africa (the STAR project). HIV testing represents an interesting case having undergone a major transformation with the introduction of innovative testing technologies like HIVST. These new testing modalities emphasise the decentralisation and demedicalisation of HIV testing, making it more accessible and user-friendly. HIV remains a significant public health concern worldwide, necessitating comprehensive strategies for prevention, treatment, care, and support (UNAIDS, 2022). While significant progress has been made in HIV testing, with approximately 85% of people with HIV globally aware of their status, approximately 15% remain undiagnosed and require access to HIV testing services. Historically, HIV testing was marked by exceptionalism due to the perception of the virus as a death sentence, resulting in practices such as mandatory testing without informed consent (Bayer et al. 2006). However, scientific advancements, particularly effective antiretroviral therapy, have transformed HIV from a life-threatening illness to a manageable chronic condition (Melo et al., 2020). This shift has influenced testing policies, emphasizing early detection, treatment as prevention, and linkage to care. Ethical frameworks for HIV testing now prioritise voluntariness and informed consent, ensuring that testing is a voluntary and informed decision made by individuals (World Health Organization 2021). We explore here instances of coercive policy in HIV testing in the context of Malawi, where HIV in the general population remains high at 7.1%, and the underlying ethical principles that may or may not support this (UNAIDS 2022).

Courts in Malawi may order individuals to go for testing if there are claims that an HIV-positive individual might have knowingly infected others (Malawi Ministry of Health, 2022). This is where we see direct coercion in HIV testing. The harm principle may be proposed as a possible justification for this mandatory testing. The harm principle, in the context of HIV testing, might hold that if an individual's behaviour puts others at risk of HIV transmission and exposure, third party intervention may be justified, including coercive measures such as mandatory testing, to prevent harm to the broader population. Legally, measures such as partner notification may also be employed without the explicit consent of the HIV-positive individual, again apparently drawing on the harm principle in policy. Yet, should it? To what extent does HIV testing in Malawi lead to a reduction in transmission, and to what extent does that relationship between testing and reduced transmission depend upon access to treatment and to preventive measures?

Indirect coercion is rather common in HIV testing, as well, due to public health practice and the relational dimension of HIV transmission. An explicit example of indirect coercion in HIV testing is in antenatal care. In this context, pregnant women are routinely tested for HIV as part of standard healthcare services, with the option to opt out. The justification for this approach might lie in both the principle of public interest and the harm principle. The argument is that identifying HIV-positive pregnant women early in their pregnancy allows for appropriate interventions, including antiretroviral therapy (ART), to be initiated promptly and minimise harm to their unborn child. ART has been shown to significantly reduce the risk of mother-to-child transmission of HIV, improving the health outcomes for both the mother and the child (Apimbaye et al. 2018). By promoting testing in antenatal care, the goal is to maximise the number of pregnant women who receive testing and subsequently reduce vertical HIV transmission to infants. By increasing the number of people who are aware of their HIV status, it is possible to reduce the overall transmission of HIV and improve health outcomes at both individual and population levels, thereby also reducing stress on the health care system, which is also in the public interest. However, ethical justification is further complicated in this case: in some cases, individuals may not fully understand the implications of the test or may feel unfairly pressured into accepting the test due to societal expectations or healthcare provider attitudes (King and Winchester 2018). For example, societal norms and expectations surrounding pregnancy and motherhood such as the expectation that all women should become mothers and should take responsibility for raising healthy children (Evens et al. 2014) can create an environment where women may feel obligated to undergo HIV testing for the well-being of their unborn child, even if they have concerns or reservations about the test. Additionally, healthcare providers, although they emphasise the voluntary nature of the test, may exert subtle pressure or influence through their demeanour, language, or the way they present the test as routine or standard care. Is this a burden on the prospective mothers that may counterweigh the ethical argument based on protecting the public interest?

In the context of HIV testing, the principle of paternalism seems to be linked to policies on the administration of pre-exposure prophylaxis (PrEP). PrEP is a preventive measure where individuals at high risk of contracting HIV take daily medication to reduce their chances of acquiring the virus (World Health Organization 2021). While PrEP has proven to be highly effective in preventing HIV transmission, it requires adherence and regular testing of HIV status to ensure its efficacy. Healthcare providers and policymakers may rely on a paternalist justification to enforce HIV testing as a prerequisite for patients accessing PrEP. Similarly, in a context of intermittent ART, such as in populations who are highly mobile, HIV re-testing is mandatory in order to reinitiate treatment (Thorp et al. 2023). Both testing and PrEP are good for the patient, as confirmation of negative HIV status is needed to ensure monitoring and effectiveness of the treatment (Zucker 2018). Whilst patients may not always be inclined to undergo testing as a pre-requisite for PrEP, some paternalistic health care providers may argue that it could be in their best interests to apply some level of coercion to ensure testing is administered alongside treatment. In this case, it may seem less controversial that this Western bioethical concept can go some way toward ethically justifying this coercive measure.

HIV prevention and control extend beyond individual concerns, as the virus's transmission has a relational dimension within sexual partnerships and communities. Policies and measures aim to increase HIV testing rates and improve access to testing services, and may include routine testing in healthcare settings, secondary distribution of testing kits, and educational campaigns. Certain testing modalities such as secondary distribution may also be coercive. Secondary distribution involves individuals providing HIV testing kits to their partners or family members, which can lead to pressure or influence on others to undergo testing, an issue raised particularly in HIV self-testing (Qin, 2016). Whilst the state does not coerce these individuals, the implementation of policies that aim at education and promoting use of testing kits can be indirectly coercive, through partner coercion. Whilst many of the ethical justifications already discussed may initially appear to adequately justify this indirectly coercive action, other factors must be considered that may render the application of these concepts inappropriate. In the contexts where gender-based violence rates are high, policies that influence individuals to coerce their partners into testing should be carefully evaluated. Gender-based dynamics can create additional strains within relationships, as introducing HIV testing may be seen as an accusation or confirmation of infidelity. Such policies may pose more harms than benefits (thus invalidating or at least counter-weighing ethical justification via the harm principle, the public interest or the duty of collective easy rescue) by exacerbating existing inequalities in the face of increasing testing uptake. When HIV testing policy is enacted within contexts of wider structural vulnerabilities, including gender inequalities, power imbalances, and high rates of interpersonal violence, these must be considered by policymakers in any policy decisions that aim to respect autonomy in HIV testing settings. It may be the case that even supposedly entirely voluntary and autonomy-respecting testing policies, such as the promotion of HIV testing through antenatal care and through secondary distribution, places women in potentially vulnerable positions where they are pressured to ‘persuade’ a partner to test. In such cases, this approach, with the onus on women to introduce testing to their partners fails to acknowledge the structural factors limiting autonomy. In the absence of a compelling argument that coercing individuals would undermine the promotion of their autonomy, coercion may be justified where the harm principle or paternalism apply. Monitoring and evaluation mechanisms should be in place to assess experiences of coercive and non-coercive policy in this context. This includes gathering feedback from individuals on their experiences and perceptions of testing as well as monitoring any instances where individuals feel coerced or pressured into testing and how this affects them. Regular review of the policy and its implementation can help identify and address any unintended consequences or ethical concerns that may arise.

In general, there is emphasis on individual autonomy in HIV testing policies. This is infringed by coercive measures, and in contexts where individual autonomy is paramount, this may provide a stronger initial argument against coercive measures. It will only be in some cases that the justifications we have explored will counterweigh infringements of liberty or autonomy where this is held as an important value by the relevant community. However, in some settings, there is less emphasis on autonomy, and coercive HIV testing policies may not face a strong initial argument against them. For example, in communities where the health and wellbeing of the population as a connected network is of key importance, decision-making surrounding individual testing and treatment ought to take into account that the individual is defined in relation to their community. In such cases, seeking individual consent may not be the appropriate approach. In addition, in states or populations where Afro-communitarianism is an accepted part of the moral framework of society, a presumption in favour of individual liberty may not be warranted, because of how the person is defined in relation to community (Chimakonam and Ogbonnaya 2022). That is not to say that different ethical traditions which may have historical roots closer to the policy context in question will always arrive at a different conclusion to analysis using Western bioethical concepts. Indeed, an emphasis on relational responsibility and a focus on community and individuality of personhood as is common in Afro-communitarian philosophy may lead to judgements in favour of HIV testing (Molefe 2019). The point is that there may be concepts from other ethical traditions than Western bioethics which ought to be considered for the ethical justification of policies.

## 4 Coercive policy and ethics in mandatory vaccination

Two of the co-authors are actively engaged in TB and HIV research in South Africa. The dual burden of HIV and TB, along with the emergence of COVID-19, presents a unique opportunity to gain insights into public perceptions of future TB and HIV vaccines and their potential implementation in the region. Vaccines are one of the most effective tools for controlling the spread of diseases like measles, diphtheria, tetanus, influenza and COVID-19. Currently, immunisations prevent 3.5 - 5 million deaths annually (World Health Organization 2023). In efforts to protect people against infection, governments and other institutions have resorted to mandatory vaccinations to increase vaccination rates, reduce the burden on the health care system, and achieve national public health goals. In May 2012, the Global Vaccine Action Plan (GVAP) was formulated and endorsed by 194 Member States at the World Health Assembly. The primary goal of this framework was to save millions of lives from preventable diseases through equitable provision of existing vaccines by the year 2020. Over the years, progress has been made with more children receiving vaccinations, countries introducing new vaccines, and the scientific research community developing newer and more effective vaccines. However, vaccine coverage has hit a plateau and even declined since the year 2020 (World Health Organization 2023).

The decline in vaccination rates has been accompanied by an alarming resurgence of measles in countries like the USA and South Africa, an effect largely fuelled by the spread of misinformation and the desire for parental autonomy pertaining to vaccination decisions. While the GVAP framework is commendable in its objectives, it makes no reference to the ethical dimensions of vaccine mandates, instead leaving the integration of vaccination objectives up to the discretion of national policymakers.

In South Africa, policymakers have historically taken a coercive approach to vaccination. In the case of childhood vaccination, parents must—in effect, if not in writing—enrol their children in the childhood vaccination programme to ensure they can later gain access to the public or private schooling system (Kling 2009). Unvaccinated children are often required by schools to update their vaccination cards as a condition of acceptance even though there is no legal requirement to do so. This situation resembles a form of indirect coercion, similar to that discussed in the case of secondary distribution of HIV tests, and is illustrative of particular structural differences between South Africa and other countries which may affect the way ethical justifications apply and whether their premises are empirically supported. While this form of coercion might be in the public interest and prevent harm to others, it raises questions relating to the balance between individual autonomy and collective wellbeing that are not highlighted in the HIV testing case. Bodily autonomy pertains to an individual’s capacity to make decisions about their own body, including how and where it is touched, how it is nourished, or otherwise affected. Most public health interventions aim not to interfere with bodily autonomy, and those that do might be considered not only coercive, but compulsive in that physical force may be used on an individual. While this is not necessarily the case with coercive policies, interventions that involve more direct bodily interference, such as injection, do carry a greater risk of physical coercion.

Mandatory vaccination has gained significant attention in the context of the COVID-19 pandemic, and in South Africa, whilst the state did not impose vaccination requirements, various other organisations took it upon themselves to do so. For instance, Goldrush Group, a gaming company in South Africa, mandated that its employees be vaccinated against COVID-19 in 2021, and subsequently terminated the employment of an individual who did not comply. The South African Conciliation, Mediation and Arbitration (CCMA) committee upheld the dismissal to be fair (Rödl & Partner 2022). The employee who brought the case to the committee relied on sections of the South African Constitution relating to the protection of bodily autonomy and psychological integrity, but the committee found that as a ‘high-risk’ individual in daily contact with colleagues, she had an obligation to be vaccinated that she had not fulfilled. The wording used by the committee aligns well with a duty of collective easy rescue, wherein an individual might have a duty to protect others from harm from a disease like COVID-19 even at a small sacrifice such as the slight infringement of their bodily integrity that is involved in receiving an injection for immunisation. As such, this case highlights the tension between individual autonomy and the duty individuals may have to help protect collective health and wellbeing, especially in the context of a highly contagious disease like COVID-19. It also underscores the role of legal and ethical considerations in shaping vaccination policies and mandates in various organisations.

Vaccine mandates can be ethically justifiable when the threat to others’ health from a particular disease is significant or where there is considered to be a public interest in people not getting sick (say, to preserve productivity at work). The duty of collective easy rescue and the protection of individuals through paternalistic action might also be referred to. However, some caveats are needed. For instance, expert confidence in safety and effectiveness of the vaccine must be high, and the expected utility of mandatory vaccination must be greater than non-coercive or non-vaccination alternatives. There must be some level of acceptance in the population of coercive measures surrounding vaccination, which may be present in the South African jurisdiction. Furthermore, the penalties or costs for non-compliance must be reasonable and proportionate (Savulescu 2021; Savulescu, Persson and Wilkinson 2020). To date, COVID-19 has claimed the lives of more than 6.8M people in less than 3 years and was for some time the leading cause of death from a single infectious agent. However, whether this significant potential harm is great enough to justify harsh penalties for non-compliance with COVID-19-controlling measures such as mandatory vaccination will depend on individual circumstances, which are in turn affected by specifics of the country in question. For instance, if the cost of non-compliance with mandatory vaccination is loss of employment, we might question whether this cost is too great in comparison to the harms that an individual risks imposing on others through transmission of the virus. The impact of loss of employment on someone will, in turn, depend on how much social or welfare support is available. Especially in contexts where alternative work may be hard to find or large groups in the population may have few savings and insecure livelihoods already, it may be inappropriate for companies to be allowed to terminate employees’ contracts because they refuse to be vaccinated. In that case, how well the ethical justification of a duty of easy rescue applies will depend on these structural factors.

That being said, Williams (2021) argues, and we agree, that selective mandates may be better warranted in settings where unvaccinated people have contact with more vulnerable people through their work or daily lives. Indeed, vaccine hesitancy has been on the rise in recent years even among health care workers, who should be among the group most informed on the efficacy of vaccines (Gur-Arie, Jamrozik and Kingori 2021; MacDonald and Dubé 2015). If one’s work involves contact with those more likely to experience severe disease, there may be more justification based on either the harm principle or the duty of collective easy rescue for the unvaccinated working individual to be required to get vaccinated. A selective mandate approach may not only protect the vulnerable, then, but also respect the individual autonomy of all those who do not pose particular risk to vulnerable others through their work or daily lives.

Limiting personal choice in favour of protecting another from harm is the common approach for justifying public health policies, as in the case of speed limits, designating public spaces as non-smoking areas, or even firing a gun in urban areas. In other words, “It is the risk of harm to others—impinging on their liberty to be safe while driving, breathe clean air, or not be shot or trampled—that makes it ethical to place limits on personal choices.” (Wynia et al. 2021). Moreover, when it comes to the vaccination of vulnerable groups, we might think that the South African government has a responsibility to protect the health of its citizens, thus mandating vaccinations for groups of vulnerable individuals like the elderly might be viewed as a legitimate exercise of state power with the justification of paternalism.

Setting aside the question of vulnerable populations, the above arguments are weaker for mandating vaccination for populations who are less susceptible to severe symptoms from COVID-19. For instance, the argument from paternalism does not apply if young people are less susceptible to COVID-19, if there are any potential harms from vaccination, as in that case, vaccination may not be in their best interest (Reiss and Caplan 2020). One objection to this point may be that without young people being vaccinated, herd immunity is much less likely to be achieved, and thus, protecting the vulnerable from harm and the healthcare system from additional stress may be much more difficult. Such arguments, however, rely on empirical claims about whether all the vulnerable can be vaccinated (thus meaning herd immunity protection is not needed). The data pertaining to how vulnerable a particular country’s population is will also affect the applicability of this ethical justification from paternalism.

Vaccine mandates are a restrictive form of vaccination policy and should ideally come in place as a last resort after other measures have failed. Alongside considering ethical justifications for enacting vaccine mandates, policymakers need to address the root causes of vaccine hesitancy in their jurisdictions, which might vary across contexts but could include the lack of efficacy of some vaccines, public mistrust in state authorities, practical difficulties with access, or past medical abuses of certain populations. Access ought to be considered as a countervailing reason against ethical justifications for vaccine mandates in some states. Mandates become unjust when certain demographic or geographic populations do not have easy access to the vaccines and are yet affected by the consequences of non-compliance to the mandates. Importantly, the level of coercion should be proportionate to the combination of these ethical considerations (Savulescu 2021).

## 5 Coercive policy and ethics in antimicrobial stewardship

One of the co-authors has conducted research on antibiotic stewardship measures in the UK [anonymised for peer review]. The UK is currently in the process of developing its next National Action Plan (NAP) on antimicrobial resistance (AMR). This builds on the previous NAP 2019-2024 (2019) and an addendum to it (2022). The addendum showed some weakening of commitments to ensuring UK policy aligns with EU policy, which is a particular issue in relation to an EU ban on use of antibiotics in groups of healthy livestock, and concerns that the UK will fall behind the EU, exacerbating AMR in the UK (Levitt 2022). It is important to examine the ethical acceptability of and justifications behind the EU ban, to judge whether the UK should re-commit to strengthening its restrictions on antibiotic use for healthy livestock in the next NAP.

The World Health Organization (WHO) introduced guidance on the production of NAPs in its Global Strategy for the Containment of Antimicrobial Resistance (2001). Since that point, 78 NAPs have been produced. Whilst the UK’s NAPs have gone hand-in-hand with EU legislation up until 2020, this was not the case after the UK’s exit from the EU. Rather, regulation of antibiotics in animal feed in the UK now relies on the UK Veterinary Medicines Regulations (2013), which does not restrict use of antibiotics in medicines or medical feed to anything near the level of the EU ban (Council of Europe 2022a; 2022b). By contrast with the UK, these EU regulations mean that farmers can no longer administer antibiotics to healthy animals as a group, only to individual healthy animals at high risk of infection. The question of whether equivalent coercive legislation should be implemented in the UK might be assessed in part by considering the ethical justifications behind the EU regulations, and whether they i) apply well in the EU context to begin with; and ii) translate well into the UK context. If the justifications presented for the coercive EU legislation are weak or invalid in that context, then they should be reconsidered altogether. If they are strong, then it remains to be seen whether the situation is equivalent in the UK such that they still apply the UK has reason to mirror the EU legislation.

The ethical justification behind the EU ban is implied in several places in policy documents, primarily in the EU’s veterinary medicinal products regulation (Council of Europe 2022a). For instance, this regulation holds that:

“Antimicrobial resistance to medicinal products for human use and veterinary medicinal products is a growing health problem in the Union and worldwide. Due to the complexity of the problem, its cross-border dimension and the high economic burden, its impact goes beyond its severe consequences for human and animal health and has become a global public health concern that affects the whole of society and requires urgent and coordinated intersectoral action in accordance with the ‘One Health’ approach.” (Section 41)

This appeal to AMR as a complex, global problem with serious consequences establishes the scale of harms that AMR could cause, and the public interest shared across the globe in addressing the problem. The statement aligns, then with ethical justification from the public interest. A public interest in maintaining antimicrobial effectiveness might justify coercion across sectors where the coercive actions protect this public interest (Johnson 2024b). Prohibiting antimicrobial use for prophylaxis in livestock in the EU is a coercive action that reduces overall antimicrobial use, and therefore can be expected to maintain effectiveness of antimicrobial drugs in the future.

Second, consider a part of the EU regulation (2022a) that implies the importance of preventing harm to others through preserving antibiotic effectiveness for human treatment. The passage reads:

“In order to preserve as long as possible the efficacy of certain antimicrobials in the treatment of infections in humans, it may be necessary to reserve those antimicrobials for humans only. It should be possible, therefore, to decide that certain antimicrobials, following the scientific recommendations of the Agency, should not be available on the market in the veterinary sector.” (Section 46)

Furthermore, the wording of another section (44) points to a possible cause of AMR through the use of antibiotics used to treat humans being administered in the livestock setting: this “*may accelerate the emergence and spread”* of resistance. We might think that if this causal link is established, then a requirement of the harm principle is fulfilled, in that harm will be prevented by state interference in farmers’ administration of antibiotics used for treating humans. If the causal link holds, then this justification based on the harm principle is indeed a strong reason for coercion via the EU ban.

However, there is a morally relevant difference between the EU and the UK contexts. Namely, voluntary action by UK farmers so far has been quite effective already, leading to a decrease in antimicrobial use in livestock in the UK of 55% since 2014 (UK Parliament 2022). The specific structural conditions of the UK mean that the ethical argument does not work in the same way: it seems that the public interest and prevention of harm to others is already served by farmers’ voluntary action in the UK, without there needing to be further restrictions put in place now. Imposing coercive action might not be the least restrictive alternative available that still effectively decreases antimicrobial use—instead, it might be unnecessarily restrictive and impose poorly harms on farmers in the UK context. As this case shows, even policy jurisdictions with many structural similarities may still have differences that change whether a policy that is ethically well justified in one jurisdiction should be applied the same way in another. Despite these both being jurisdictions in which it may appear very appropriate to rely on a Western ethical tradition, there are limitations to the ethical concepts none-the-less, and these ought not be assumed away due to similarities between the EU and UK.

## 6 Discussion

In this final section, we reflect on what case studies of policy across different contexts and interventions—which sometimes may employ similar Western ethical justifications for coercion, but in some cases ought not—can tell us about the ethical justifiability of coercive measures in public health policy.

The ethical justifications we have considered here are a part of the Western ethical tradition. They have inbuilt limitations according to the premises of their arguments—for instance, requiring evidence of not-too-great a harm to the individual being prevented from acting in order to ensure they don’t harm another. Whether these premises are true will depend on structural context, and as such, our case studies highlight how context affects the strength of these Western ethical justifications. At the same time, we can question the extent to which the justifications are the most appropriate choices at all, in global contexts where different political and economic conditions, different cultures and moral frameworks, prevail. If the most ethically appropriate justifications should be used to justify coercive public health policy in a particular jurisdiction, then we need to recognise both the limited strength of Western bioethics concepts in a general way depending on context specifics, and what may be their further limited applicability in comparison to ethical traditions arising in the policy jurisdiction. For instance, some African bioethics concepts may be more appropriate and more applicable in the context of policymaking on HIV testing in Malawi, and may face fewer of the weaknesses that Western bioethics concepts face in that context. This is certainly not always the case: some concepts from Ubuntu ethics, for instance, may provide strong ethical justifications for coercive policy choices in Australia, and some concepts from Aristotelian virtue ethics may provide strong ethical justification for coercive policy choices in Brazil. The point is that we ought not universalise from a Western bioethical tradition without analysing the applicability of these ethical concepts, in *whatever* policy jurisdiction we are assessing.

What our case studies highlight is the complexity of ethical justifications for coercion, and how this interacts with context. Ethical considerations regarding coercion are highly context-specific and deeply linked to certain empirical and moral assumptions. The idea that individuals have moral obligations toward others and the nature of this obligation relies on specific conceptions of the individual in relation to the community and to others, and the way this is presumed within, for instance, the duty of collective easy rescue, shows the influence of Western moral norms on ethical justifications that have been proposed to apply across contexts in public health ethics. Ethical frameworks can vary significantly in their emphasis on individual rights, collective wellbeing, and the nature of duties. For example, the harm principle focuses on whether they have duties to other individuals alone whilst the duty of collective easy rescue might reflect responsibility towards a group.

Our first case study of HIV testing in Malawi highlights how these duties might be coloured by social norms and expectations, and how they might be more or less legitimately applied in that context depending on whether people’s decisions are wrongly influenced by others, including partners, where unfair power relations apply. Obligations toward communities, by contrast, might be seen as more or less strong reasons for testing depending on how important the community is in the moral and cultural fabric of a state’s population. That will also be affected by structural conditions that influence how much of a burden is posed by measures to protect a community, or how much state support is available. It may be that, on the whole, due to the limitations of Western bioethics concepts, individual-centred ethical justifications are less appropriate than Ubuntu community-centred ethical concepts for analysing that might better situate testing in the community context, or concepts from other ethics traditions appropriate to the Malawi context.

As our second case study of vaccination mandates in South Africa demonstrates how even where a state does not enforce certain visions of collectives at workplaces or in health care environments, corporate and institutional actors may take such decisions themselves, which might also require ethical reflection by states. This raises questions about who should define communities and agents in policymaking, and the responsibility individuals bear in safeguarding the health and wellbeing of the vulnerable, which may also vary according to cultural norms surrounding respect for the elderly and other factors such as history of vaccination and the epidemiological landscape of a jurisdiction.

Finally, questions about the differences between states and what makes them legitimate in the eyes of their publics might come to the fore in determining the appropriateness of ethical justifications for coercion. In the UK, there may be less acceptance in this liberal democratic society of action by the state that is not the least restrictive alternative, as highlighted by our UK AMR NAP case study. This may be supported by economic conditions that allow farmers more support for voluntarily enacting measures that may cost them some profit.

Context-specific factors like these are highlighted through case studies of coercive interventions, and ought to make us wary of generalising ethical justifications for coercive action across contexts without adequate further reflection.

## Conclusion

In this paper, we have examined ethical justifications for coercive public health measures. We have assessed how ethical concepts including the harm principle, a collective duty of easy rescue, paternalism, and the public interest might feature as ethical justifications for public health policies. The policies we have discussed in our case studies span across HIV testing, vaccination, and antibiotic use restrictions in livestock. The contexts we have considered span across Malawi, South Africa, and the UK. In reflecting on ethical justifications and their limitations within and outside particular geographic, economic, political, moral, and social contexts, we highlight how important it is that coercive policy is considered not through unreflective universalisation of Western ethics concepts, but rather with a thoughtful approach to factors that may affect the strength and validity of proposed ethical justifications for coercive public health policies.

## Footnotes

2 It should be noted that while this presumption is common, it is by no means uncontroversial. Among those who question a presumption in favour of liberty, Dawson (2016) usefully highlights how some analyses of coercive policies fail to weigh liberty *against other values*. Outside liberal democratic settings, this value assumption may be even less justified, particularly in states where authority, hierarchy, and community play a larger role in decision-making, which might contravene individual liberty and autonomy in a way that aligns with the moral framework of that state’s population.
